# Nile tilapia and gilthead seabream dietary self-selection of alternative feeds

**DOI:** 10.1007/s10695-024-01373-y

**Published:** 2024-07-10

**Authors:** Rodrigo Mendes, Luís E. C. Conceição, Jorge Dias, Sofia Engrola, Francisco J. Sánchez-Vázquez

**Affiliations:** 1 Departamento de Fisiología, Facultad de Biología, Universidad de Murcia, 30003 Murcia, Spain; 2 Sparos Lda, Área Empresarial de Marim, Lote C, 8700-221 Olhão, Portugal; 3 Centre of Marine Sciences, (CCMAR/CIMAR LA), Universidade Do Algarve, Campus de Gambelas, 8005-139 Faro, Portugal

**Keywords:** Animal behaviour, Fish physiology, Self-selection, Alternative feeds, Nile tilapia, Gilthead seabream

## Abstract

Classical assessments of new fish feeds are anthropocentric, focusing mainly on growth. Although this methodology is accurate, it does not consider the fish’ perspective. This study aimed to investigate the behavioural responses and feed preferences of Nile tilapia (*Oreochromis niloticus*) and gilthead seabream (*Sparus aurata*) through a self-selection trial using self-feeders. Both species were offered three feeds: a control (PD) commercial-like feed and two diets (ORG1 and ORG2) formulated with different inclusions of alternative ingredients to address some of the current environmental concerns and/or ethical issues often associated with commercial formulations. Three groups of tilapia with an average weight of 163.0 g ± 4.3 g (mean ± SD) and four groups of seabreams with 174.7 g ± 27.0 g were tested. Tilapia exhibited a preference for ORG2 (46.5%), influenced by the sensory properties of the feed and post-ingestion signals. Seabream did not show a preference for any feed. These findings highlight the effectiveness of self-selection experiments in allowing fish to express their feeding behaviour and preferences. Therefore, this approach should be considered in the initial screening and design of new aquaculture feeds and ingredients.

## Introduction

In the wild, since no single feed supplies all essential nutrients, most fish show dietary selection and pick up different items to create a complete and balanced diet according to their physiological needs to survive (Huntingford 2020). Fish are able to select and regulate the intake of macronutrients and energy through a process known as “nutritional wisdom” (Luz et al. 2018; Raubenheimer and Simpson 1997; Simpson and Raubenheimer 2001). In order to restore the metabolic balance as a result of a nutritional challenge, “specific hungers” have the ability to sense and ingest particular nutrients and/or substances in diets (White et al. 2000). Therefore, fish select their feed based on a series of complex regulatory mechanisms, associated with physiological, learning, and behavioural processes, involving hormonal and neural activities in the brain, gastrointestinal tract, and liver (Comesaña et al. 2020; Forbes 2001; Fortes-Silva et al. 2016; Otero-Rodino et al. 2016; Richter 1943; Simpson and Raubenheimer 2001). Accordingly, fish feeding behaviour is a relevant characteristic that should be considered when farming aquaculture species.

Aquaculture plays a major role in society by providing to the growing world population a vital source of animal protein; however, its development can be hampered by its feeds (FAO 2022). Although aquafeeds have been commonly based on marine (e.g., fishmeal and fish oil) and plant-based sources (e.g., soy), these ingredients often encompass questionable environmental implications (e.g., resource consumption, global warming) that concern consumers (Hilmarsdóttir et al. 2022; Little et al. 2016). The inclusion rates of these ingredients have dropped considerably over the past years, and varying degrees of success in reducing their environmental impacts have been achieved (Colombo et al. 2022; Glencross et al. 2024; Little et al. 2016). Nevertheless, research has focused on finding more alternative ingredients (e.g., single-cell microorganisms (bacteria, cyanobacteria, microalgae and yeast) and non-traditional plant meals (sunflower, quinoa, rapeseed, lupins)) that could address societal demands and potentially reduce some of the negative environmental impacts of the sector (Glencross et al. 2024; Hilmarsdóttir et al. 2022; Newton et al. 2023). Therefore, when developing feeds with alternative ingredients, it is necessary to address their effects on fish.

The classic approach to investigate the effects of new diets occurs through growth experiments mainly based on physiological mechanisms; however, solely this method might not totally reveal the full picture (Roy et al. 2020). Although this methodology is accurate and have yielded considerable knowledge on animal production, it has several disadvantages (e.g. time-consuming, expensive) and does not consider fish preferences (Brännäs and Strand 2015; Filho et al. 2018; Fortes-Silva et al. 2016). Conversely, self-selection methods allow fish to freely and voluntarily accept the given diet, while taking into account fish feeding behaviour and learning processes (Fortes-Silva et al. 2016). Furthermore, this methodology allows fish to choose which feeds better suits their nutritional and energetic needs (Fortes-Silva et al. 2016). Additionally, it can also be used to investigate the detection and acceptance/rejection of feed additives, toxic substances and antinutritional factors (e.g. as phytic acid and phytate) (Costa et al. 2022; Fortes-Silva et al. 2016). Therefore, to provide a more complete perspective on the potential of novel diets, growth experiments could be complemented with self-selection methods.

Self-feeders have proven to be useful to investigate fish feed intake regulation and dietary preferences. This methodology allows fish to evaluate and sense the organoleptic properties (e.g., taste, texture, smell), nutritional and ingredient composition of the feeds (Filho et al. 2018; Fortes-Silva et al. 2016; Raubenheimer et al. 2012). Self-feeders have been used and validated for Nile tilapia (*Oreochromis niloticus*) and gilthead seabream (*Sparus aurata*), but also for several other species, such as goldfish (*Carassius auratus*), sharpsnout seabream (*Diplodus puntazzo*) and rainbow trout (*Oncorhynchus mykiss*) (Atienza et al. 2004; Fortes-Silva and Sánchez-Vázquez 2012; Montoya et al. 2012; Pratiwy and Kohbara 2018; Puchol et al. 2022; Sánchez-Vázquez et al. 1998; Yamamoto et al. 2001). However, knowledge about fish behaviour and its controls have not yet been totally understood and should be further explored, especially when considering the initial screening and design of potential new aquaculture feeds (Fortes-Silva et al. 2012; Pratiwy and Kohbara 2018; Puchol et al. 2022).

The present work aimed to investigate the acceptability, behavioural response, feed intake regulation and dietary preferences of two commercially important fish species—freshwater (Nile tilapia) and marine (gilthead seabream)—using self-feeders, to test the acceptance of non-conventional diets.

## Materials and methods

### Formulation and analysis of the diets

Three experimental diets (PD, ORG1 and ORG2; pellet size, 4 mm) for each species (Nile tilapia and gilthead seabream) were formulated and produced by SPAROS Lda (Olhão, Portugal). A control diet (PD) was formulated to mimic current commercial feeds. The remaining two diets (ORG1 and ORG2) were formulated to include alternative non-traditional ingredients (e.g. single-cell microorganisms, sunflower, quinoa, rapeseed, lupins) to address some of the current environmental concerns and/or ethical issues often associated with ingredients present in traditional commercial formulations. The ingredient selection (Tables 1 and 2) was chosen based within an organic framework (ingredients that can be found on the market as organic), on market availability and nutritional composition. The inclusion levels were adjusted for each species, according to existing knowledge on tolerance to different ingredients as well as their nutritional and especially amino acid requirements, without compromising fish growth, development, and welfare. Initially, a pilot-scale twin-screw extruder (CLEXTRAL BC45, France) equipped with a screw diameter of 55.5 mm was used to manufacture the feeds. A temperature range of 105–110 °C was used for the extrusion process. All batches of extruded feeds were dried in a convection oven (OP 750-UF, LTE Scientifics, United Kingdom).

**Table 1:** Diet formulation (% inclusion levels) and proximate composition (% as fed) of the experimental diets (PD, ORG1 and ORG2) for Nile tilapia (*Oreochromis niloticus*)

Ingredients (% inclusion levels)	PD	ORG1	ORG2
Fishmeal	5.00		
Poultry meal	5.00		
Brewer's yeast	5.00	10.00	10.00
*Spirulina*		3.50	7.00
Pea protein concentrate		5.50	2.25
Wheat gluten		3.50	7.00
Corn gluten meal	11.60		
Soybean meal	18.00	18.00	
Rapeseed meal	6.50	6.50	13.00
Sunflower meal	3.25	7.50	15.00
Wheat meal	27.75	6.70	9.35
Rice bran full fat	10.00	10.00	10.00
Corn meal		6.95	6.95
Quinoa		5.00	5.00
Faba beans		9.00	7.00
Vitamin and mineral premix	1.00	1.00	1.00
Choline chloride	0.20	0.20	0.20
Antioxidant powder (Verdilox)	0.20	0.20	0.20
Mono-calcium phosphate	1.80	2.15	2.05
L-Lysine	0.30		
DL-Methionine	0.10		
Fish oil	0.90	1.00	1.00
Soybean oil	3.40	3.30	3.00
Proximate Composition (% as fed)	**PD**	**ORG1**	**ORG2**
Dry matter (DM)	92.10	91.74	90.58
Ash	6.27	6.33	6.22
Crude protein	33.50	34.06	33.75
Crude fat	8.43	7.63	6.75
Gross energy (kJ/g^−1^)	19.33	19.24	18.18

All values are reported as mean of duplicate analyses

**Table 2:** Diet formulation (% inclusion levels) and proximate composition (% as fed) of the experimental diets (PD, ORG1 and ORG2) for gilthead seabream (*Sparus aurata*)

Ingredients (% inclusion levels)	PD	ORG1	ORG2
Fishmeal super prime	20.00	20.00	20.00
Poultry meal	10.00		
Brewer's yeast		5.00	5.00
*Spirulina*		3.50	7.00
Pea protein concentrate		16.50	11.50
Wheat gluten			3.00
Corn gluten meal	8.00		
Soybean meal	16.00	16.00	
Rapeseed meal	3.30		3.30
Sunflower meal	6.00		10.00
Wheat meal	5.90	5.55	
Wheat bran		5.00	5.00
Quinoa		5.00	5.00
Faba beans	7.00	7.00	7.00
Whole peas	7.00		7.00
Vitamin and mineral premix	1.00	1.00	1.00
Choline chloride	0.20	0.20	0.20
Antioxidant powder (Verdilox)	0.20	0.20	0.20
Mono-calcium phosphate	0.70	1.05	1.00
L-lysine	0.30		
DL-methionine	0.10		
Fish oil	4.60	4.60	4.60
Soybean oil	9.70	9.40	9.20
Proximate composition (% as fed)	**PD**	**ORG1**	**ORG2**
Dry matter (DM)	94.22	95.11	91.50
Ash	7.07	6.36	6.33
Crude protein	40.91	43.03	41.01
Crude fat	18.43	16.63	15.22
Gross energy (kJ/g^−1^)	22.20	22.47	22.00

All values are reported as mean of duplicate analyses

Feed samples were grounded and analysed for dry matter (105 °C for 24 h), gross energy in an adiabatic bomb calorimeter (Werke C2000, IKA, Germany), crude protein by the Kjeldahl method (automatic flash combustion; Leco FP-528, Leco, St. Joseph, USA) (N × 6.25%), lipid content by diethyl ether extraction (Soxtherm Multistat/SX PC, Gerhardt, Königswinter, Germany; 150 °C) and ash by heating in a muffle furnace (Nabertherm L9/11/B170, Germany) at 450 °C for 24 h. All diets were formulated to be isonitrogenous (crude protein of ~ 33.8% and 41.7% as fed, for tilapia and seabream, respectively) and isoenergetic (gross energy of ~ 18.9 kJ/g and 22.2 kJ/ as fed, for tilapia and seabream, respectively) (Tables 1 and 2).

The total amino acid content of the experimental feeds was determined through a series of analytical procedures. Initially, samples underwent hydrolysis in aqueous hydrochloric acid. For cysteine, cystine, and methionine, prior oxidation with hydrogen peroxide and formic acid at cold temperature was performed. Following this, sample pH adjustment, volume adjustment with loading buffer, and filtration were carried out. Amino acid separation occurred using an amino acid analyser, with detection facilitated through post-column derivatization with ninhydrin reagent and measurement at wavelengths of 440 nm and 570 nm. Tryptophan quantification involved high-performance liquid chromatography (HPLC), with preliminary exposure to alkaline hydrolysis. Separation on AAA occurred via a sodium cation exchange column, with post-column derivatization using O-Phtahalic aldehyde (OPA) and detection through fluorescence at wavelengths of 338/425 nm. Amino acid profiles of the experimental diets given to both species are presented in Tables 3 and 4. Although in ORG1 the diets exhibited lower methionine levels, the amino acid requirement was fulfilled.

**Table 3:** Amino acid profile (g/100 g fed basis) of the experimental diets for Nile tilapia (*Oreochromis niloticus*)

Amino acids (g/100 g fed basis)	PD	ORG1	ORG2
Arginine	1.77	2.19	2.01
Histidine	0.74	0.77	0.76
Lysine	1.79	1.77	1.46
Threonine	1.24	1.25	1.27
Tryptophan	0.36	0.41	0.40
Isoleucine	1.32	1.36	1.30
Leucine	3.11	2.42	2.36
Valine	1.55	1.58	1.60
Methionine	0.75	0.52	0.59
Phenylalanine	1.62	1.55	1.53
Cysteine + cystine	0.52	0.53	0.54
Tyrosine	1.14	1.16	1.12
Aspartic acid	2.68	2.98	2.54
Glutamic acid	6.46	6.32	6.95
Alanine	1.95	1.55	1.60
Glycine	1.63	1.48	1.54
Proline	2.22	1.93	2.04
Serine	1.62	1.61	1.60

All values are reported as mean of duplicate analyses

**Table 4:** Amino acid profile (g/100 g fed basis) of the experimental diets for gilthead seabream (*Sparus aurata*).

Amino acids (g/100 g fed basis)	PD	ORG1	ORG2
Arginine	2.80	2.75	2.69
Histidine	0.93	1.01	0.94
Lysine	2.69	2.84	2.55
Threonine	1.59	1.61	1.63
Tryptophan	0.47	0.49	0.50
Isoleucine	1.62	1.78	1.68
Leucine	3.36	3.15	2.96
Valine	1.97	2.07	2.08
Methionine	0.96	0.74	0.92
Phenylalanine	1.88	1.88	1.77
Cysteine + cystine	0.52	0.51	0.49
Tyrosine	1.39	1.42	1.34
Aspartic acid	3.69	4.31	3.74
Glutamic acid	6.63	6.66	6.76
Alanine	2.41	2.17	2.15
Glycine	2.36	2.00	2.02
Proline	2.18	1.74	1.82
Serine	1.81	1.88	1.84

All values are reported as mean of duplicate analyses

### Fish and husbandry conditions

Nile tilapia (*Oreochromis niloticus*) were provided by the University of Murcia from a mono-sex male population (offspring tilapia, GMT®). The experiment was carried out in the chronobiology laboratory at the University of Murcia. At the start of the study, 33 fish were randomly distributed in three homogeneous groups (CV < 4%), with an average initial body weight of 163.0 g ± 4.3 g (mean ± SD), into indoor fiberglass tanks of 300 L in a recirculating aquaculture system (RAS). Each tank contained 11 tilapia and was equipped with a protein skimmer, as well as mechanical, biological, UV filtered and aerated water. Fish were allowed to acclimate to laboratory conditions for at least 2 weeks, during which time they were fed a commercial diet (Skretting TI-3 (3.2 mm); with % DM: 32.0% crude protein, 6.0% crude fat and 5.8% crude fibre), which was supplied by hand until visual satiation once a day. Abiotic parameters, feed intake and mortality were measured and recorded daily. A photoperiod of 12 h:12 h (09 h 00 to 21 h 00 lights on) light/dark period was maintained during the study. Average water temperature was 29.0 ± 1.0 °C, pH of 7.2 ± 0.2, dissolved oxygen of 6.9 ± 0.4 ppm and ammonic nitrogen of 0.7 ± 1.0 mg/l. The experiment lasted for 36 days.

Gilthead seabream (*Sparus aurata*) were provided by IMIDA from San Pedro del Pinatar (Spain). The experiment was performed at the Aquaculture Laboratory located in Algameca (Cartagena, Spain). Four groups (CV ~ 15%) of 8 fish with an average initial individual weight of 174.7 g ± 27.0 g were maintained in indoor fiberglass tanks of 150 L in a in a flow-through system. Each tank was equipped with a protein skimmer, as well as mechanical, biological, UV filtered and aerated water. Fish were allowed to acclimate to laboratory conditions for at least 2 weeks, during which time they were fed a commercial diet (Skretting L-4 Alterna 2P; with % DM: 46.5% crude protein, 20.0% crude fat and 3.4% crude fibre), which was supplied by hand until visual satiation once a day. Abiotic parameters, feed intake and mortality were measured and recorded daily. The animals were kept with a photoperiod of 12 h:12 h (09 h 00 to 21 h 00 lights on) light/dark period at an average water temperature of 27.0 ± 1.0°, salinity of 37 ± 1.0 ppm, pH of 7.6 ± 1.0 for pH and dissolved oxygen of 6.3 ± 0.5 ppm. The experiment lasted for 67 days.

### Experimental setup

**Fig. 1:**
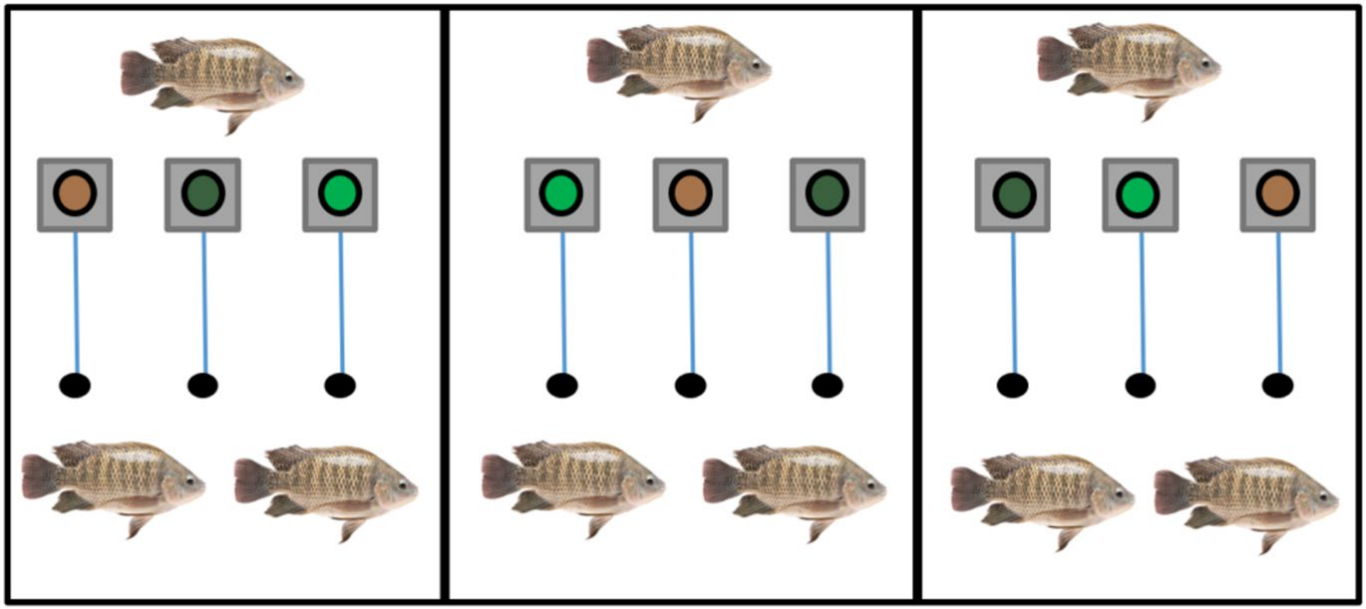
Experimental setup using Nile tilapia (*Oreochromis niloticus*). Each of the three feeders inside each tank, contained a specific feed (PD, ORG1 and ORG2)

**Fig. 2:**
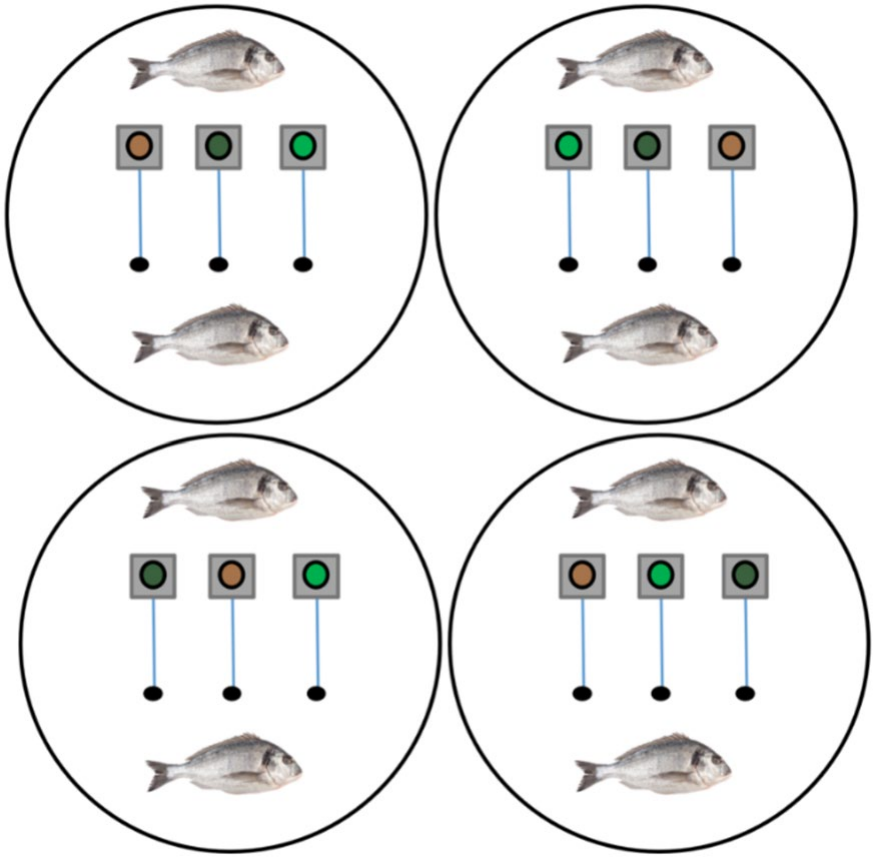
Experimental setup using gilthead seabream (*Sparus aurata*). Each of the three feeders inside each tank, contained a specific feed (PD, ORG1 and ORG2)

The experimental setup of both experiences is present in Figs. 1 and 2. The experiments were performed in accordance with Fortes-Silva et al. (2012). Three self-feeders provided by the University of Murcia were equipped in each tank. The position of each diet (PD, ORG1 and ORG2) on the feeders also varied between tanks, to avoid a possible positional effect. The feeding systems were connected with an electric transformer (one for five self-feeders). Each of them was composed of a trigger (a switch with rubber tip), actuated by the fish, placed 2 cm above the water surface, connected to an electromagnet and a feeder (EHEIM 3581, Deizisau, Germany) that delivered a predetermined amount of feed (1 pellet = 0.04 g) after each trigger actuation and electromagnet activation. To determine the daily intake, every day the feed remaining in the feeder was weighed at the same time (11:30) and subtracted with the total number of grams given the previous day, before refilling the feeder recipient for the next day. After percentages of the offered diets exhibited a statistically significant difference for one feed, diets were switched between feeders to provide a challenge for the fish, reduce the possible preference and influence for a particular string sensor or relative position of the self-feeders. In the case of seabream, on day 50, a fasting period of 10 days started as a challenge test to motivate the fish to choose a diet.

### Data analysis and statistics

The statistical analysis was performed with the IBM SPSS software, version 23.0. The experimental unit considered was the tank (*n* = 3 for tilapia and 4 for gilthead seabream). The relative selection of each diet was expressed as a percentage of the total feed consumed, considering the total diets as 100%. The feed intakes were expressed as total grams of feed ingested/% of body weight. *Arcsine* transformations of feed intake percentages were performed. In the days where a specific diet was significantly selected, the percentages of feed consumed were compared by one-way ANOVA, followed by a Tukey’s post hoc test to examine significant pair-wise comparisons, before meeting criteria for normality and homogeneity using Shapiro–Wilk and Levene’s test, respectively. The statistical significance was considered at *P* < 0.05. 

## Results

**Fig. 3:**
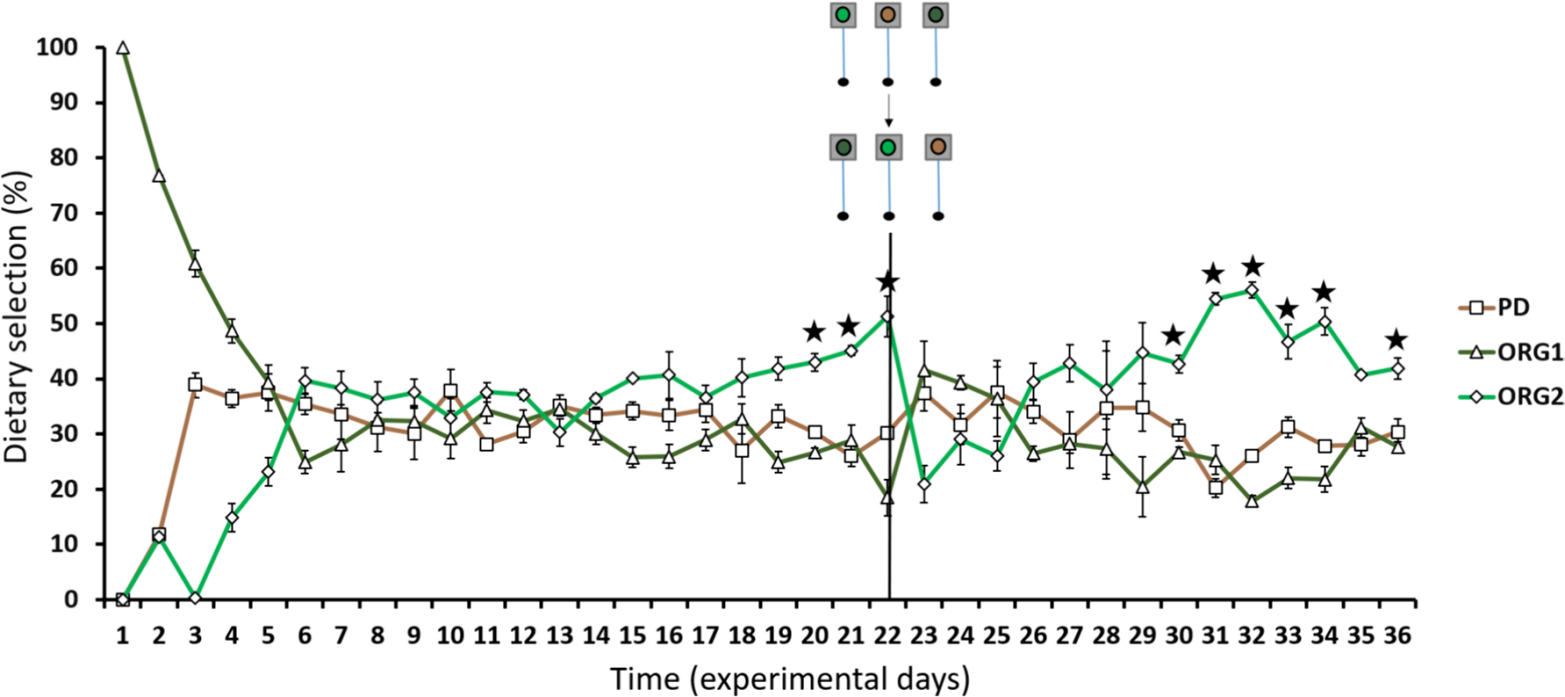
Evolution of average daily intake (% total grams of feed ingested) of three diets (PD, ORG1 and ORG2) by Nile tilapia over 36 days. Diets were changed between feeders on day 22. Lines represent the mean counts ± SD (*n* = 3 tanks). Stars represent significant differences (one-way ANOVA, *p* < 0.05)

Nile tilapia reached a final body weight of 194.7 ± 3.9 g, all fish survived, and on average feed consumed daily represented 0.75% of average body weight/day. Uneaten and wasted feed was negligible, only around 2% of the total given feed; thus, the amount of feed demanded by the fish was almost entirely ingested. The dietary preference (Fig. 3) in self-feeders initially demonstrated an adaptation period to the feeders of around 5 days. During this time, fish preferred the position of specific feeders, rather than the feed itself, but quickly changed their behaviour. All diets were chosen similarly for several days before an increase in preference for diet ORG2 was observed during three consecutive days (with an average of 46.5%; *p* < 0.05). Throughout the same period diets, PD and ORG1 were preferred on average 28.9% and 24.7%, respectively. After diets were switched between feeders on day 22, another period of equal preference remained, while from day 30 until the end of the experiment, diet ORG2 was once again mainly chosen (between 40.7% and 56.0%; *p* < 0.05).

**Fig. 4:**
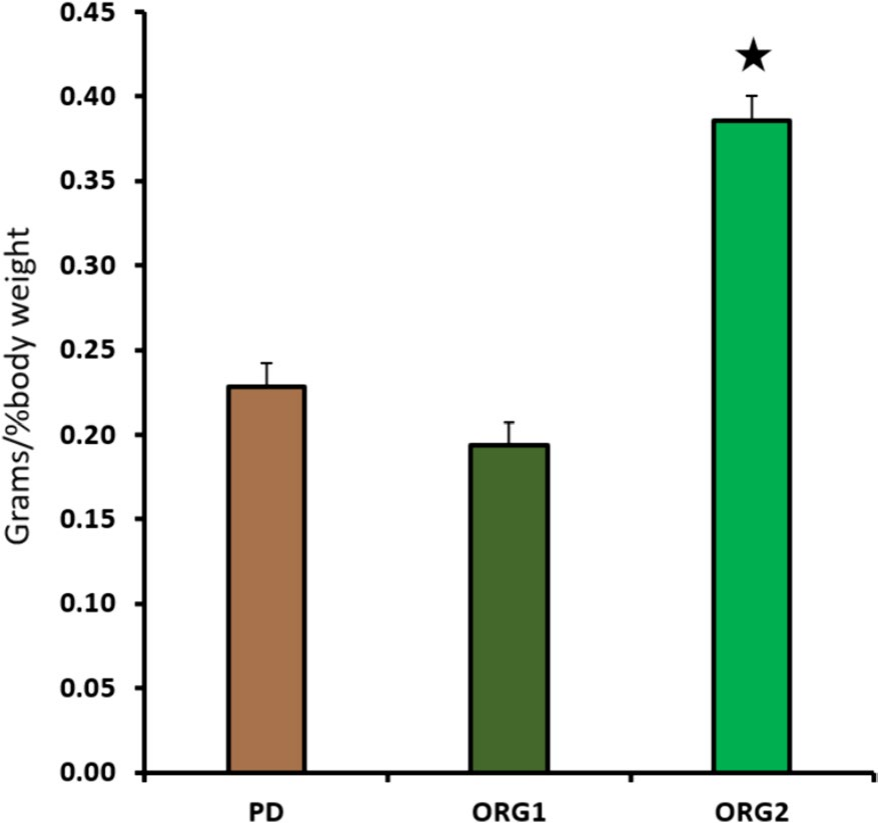
Average daily intake (total grams of feed ingested per percentage of body weight) of three diets (PD, ORG1 and ORG2) by Nile tilapia only during days with statistically significant differences. Bars represent the mean counts ± SD (*n* = 3 tanks). The star represents significant differences (one-way ANOVA, *p* < 0.05)

Diets PD (0.24 g/% BW) and ORG1 (0.21 g/% BW) were consumed 38.5% and 46.2%, respectively, less (*p* < 0.001) than diet ORG2 (0.39 g/% BW) during days with statistically significant differences (Fig. 4).

**Fig. 5:**
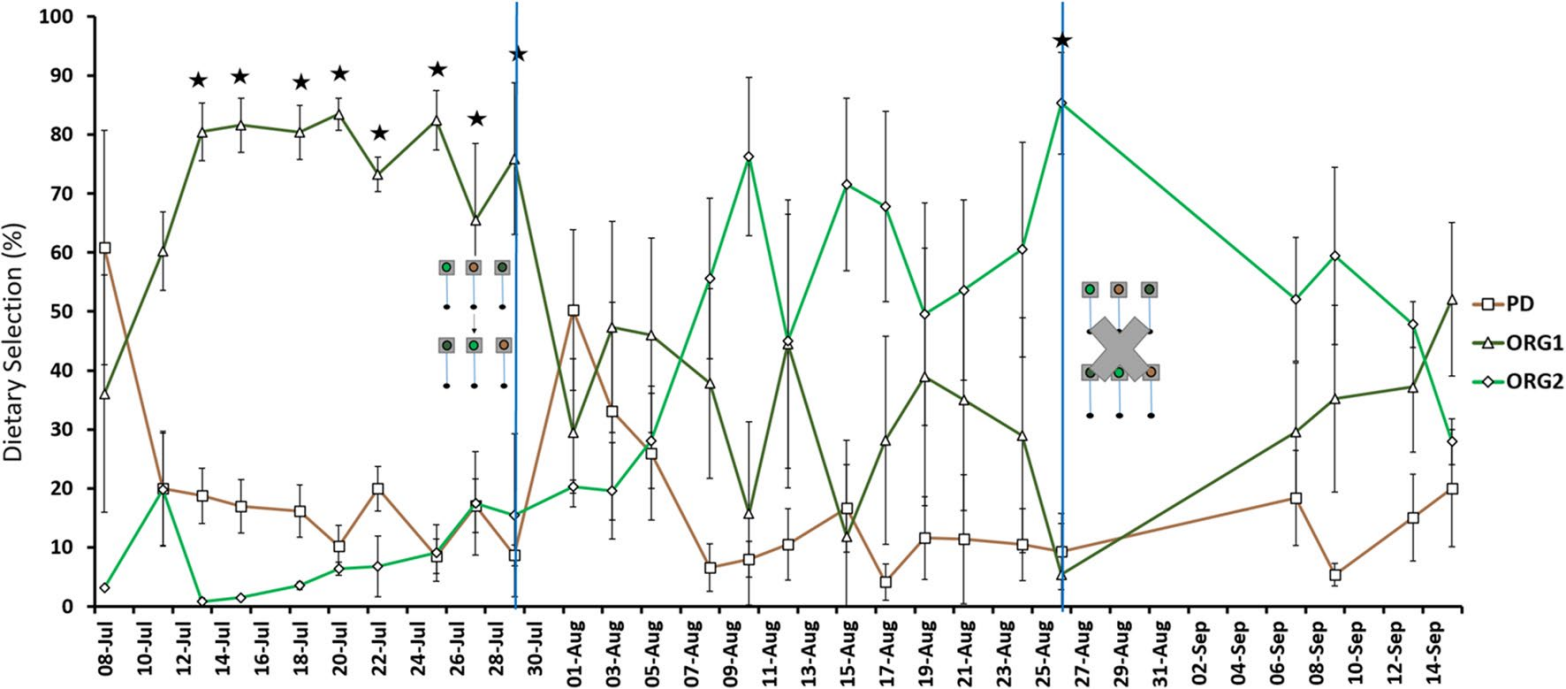
Evolution of average daily intake (% total grams of feed ingested) of three diets (PD, ORG1 and ORG2) by gilthead seabream over 67 days. Diets were changed between feeders on day 22. Fish were fasted for 10 consecutive days starting on day 50. Lines represent the mean counts ± SD (*n* = 4 tanks). Stars represent significant differences (one-way ANOVA, *p* < 0.05)

Gilthead seabream final weight was 264.4 ± 29.5 g, no mortality was recorded, and average feed consumption was 1.21% of the animal’s biomass. Compared to tilapia, seabream wasted slightly more feed (< 5%). From the initial days of the study, fish exhibited a clear preference for diet ORG1 (between 65.50% and 83.45%; *p* < 0.05). However, after diets were switched between feeders, the preference for diet ORG1 fell, while for diet PD and ORG2 increased (Fig. 5). For several days, no statistically significance was achieved. Then, fish were fasted for 10 days, starting on day 50, as a challenge test to motivate them to choose a diet, according to Aranda et al. (2001). Nevertheless, even after this approach, a consistent preference was not achieved (*p* > 0.05).

## Discussion

Fish can choose which feed items to ingest mainly based on size, palatability, ingredients and proximal composition (Raubenheimer et al. 2012). Fish are also able to identify and evaluate distinct amino acid profiles between diets (Fortes-Silva et al. 2012). All feeds were formulated to contain the minimum requirements of every essential amino acid (EAA). However, diet ORG1 presented the lowest levels of methionine. Although methionine was near the lowest requirement, it was enough to fulfil the species physiological state for normal growth (NRC 2012). Nevertheless, it is possible that this decrease in methionine could have affected fish dietary choice, particularly in tilapia, coupled with diet palatability and ingredient composition, as all feeds had the same size, were isonitrogenous and isoenergetic.

Tilapia consistently preferred ORG2 feed, influenced by learning-reward behaviour, post-ingestion signals and the orosensory properties of the diet. In a population, there may be only one dominant fish that is curious enough to pull the trigger, but if rewarded, this information may be socially transmitted, learned and repeated by all individuals (Millot et al. 2014). On the initial days of the experiment, tilapia showed a preference for one feeder, which changed after some time after assessing the content of the other feeders, demonstrating their exploratory and learning behaviour, as shown by Figueiredo et al. (2023). A similar situation was recorded using European seabass (*Dicentrarchus labrax*), which exhibited a preference for one of the self-feeders (Aranda et al. 2000). Nile tilapia chose diet ORG2 with an intake 0.75% of fish weight/day, and most pellets were consumed (less than 2% of the total given feed was wasted). Similarly, Fortes-Silva et al. (2012) reported a negligible food waste of 1% with tilapia. Pratiwy et al. (2018) tested the growth performance of Nile tilapia reared under self-feeding systems and showed feed intake values of around 1.85%/body weight. Fish had to evaluate the quality and nutritional composition of the feeds, based on a wide range of physiological processes. Accordingly, the choice for diet ORG2 was presumably based on tilapia nutritional needs (post-ingestive and/or post-absorption) coupled with feed organoleptic characteristics (texture, flavour and odour), which after evaluation, was probably more able to satisfy their physiological state (Brännäs and Strand 2015; Fortes-Silva et al. 2016). Likewise, other studies with European seabass and tilapia reported a similar behaviour (Fortes-Silva et al. 2016, 2012; Rubio et al. 2006). It is important to note since fish required almost three weeks to exhibit a preference and no feed was predominantly chosen from the beginning of the study; it can reflect the less clear differences between feeds. In a study by Carlberg et al. (2015), Arctic charr (*Salvelinus alpinus*) took 9 days to define a pattern. Fortes-Silva et al. (2010) noted that tilapia clearly preferred, since the beginning of the experiment, diets containing phytase and that after switching feeds, the pattern was re-established only after 3 days. In the present study, after feed was switched between feeders, tilapia also resumed and sustained the previous pattern of selection of diet ORG2, while maintaining a constant consumption of other diets, meaning that the fish established levels of consumption for each feeds. However, once again they took some time (9 days), pointing out the effect of the minor differences between the diets. These findings are in accordance with Fortes-Silva et al. (2010), who reported that a diet with 1500 IU kg^−1^ phytase was preferred throughout the trial, even after switching feeders. Conversely to tilapia, gilthead seabream was not able to select a feed.

Gilthead seabream did not show a consistent preference for any particular feed, which can be due to several factors. The experiment was performed with rapidly growing juveniles at a high water temperature during summer. Since it was a flow-through aquaculture system, in this scenario, the homeostatic system, which is associated with high energetic demands, may have overridden the hedonic regulation of feeding behaviour, preventing seabream from efficiently discriminating between diets (Puchol et al. 2022). Another possible explanation for the lack of diet discrimination is because all three experimental diets were nutritionally similar to the previously fed commercial feed, meaning that fish were familiar with it and did not notice enough differences (Pulido-Rodriguez et al. 2021). There was a higher variation on the daily intake of feeds compared with tilapia, which could be related with the more curious and aggressive behaviour of seabream towards feed (Puchol et al. 2022). There are few studies available regarding seabream using self-feeders. Nevertheless, it was shown that fish with around 250 g could select a diet with distinct oxidation levels of dietary lipids after 10 days with a preference of 82% and 7 days after switching feeders with an average intake of 1.57%/body weight (Montoya et al. 2011). Similarly to what occurred with tilapia, it is possible that on the initial days, seabream were preferring a specific feeder on each tank that, by coincident, contained diet ORG1. A specific feeder was also selected on the initial experimental days with European seabass and gilthead seabream by Aranda et al. (2000) and Montoya et al. (2011), respectively. Therefore, it was necessary to change the positions of the feeders to assert dietary preferences and avoid any preference for a specific position as it was noted by Puchol et al. (2022). Indeed, after changing the position of the feeders, seabream decreased their intake for ORG1 and never achieved a clear preference for any of the given feeds (Montoya et al. 2011). Montoya et. al. (2011) observed two selection patterns after changing the position of the feeders: Some fish groups resumed their selection for a specific diet, while the other groups did not show a clear preference for any diet until they were subjected to a 3-week fasting period. In the present study, seabream were also fasted, during 10 days, aiming to define a feeding pattern as the physiological state of fish caused by oxidative stress due to fasting would reinforce their selection behaviour (Montoya et al. 2011). However, seabream were not observed to define a preference, as the compensatory bite activity increase was not enough. Conversely, after a fasting period of 6 and 15 days, European seabass increased the intake for diets richer in protein and energy to recover their metabolic status (Aranda et al. 2001; Vivas et al. 2003). Although there was not a defined pattern, the general performance of the fish was not a concern.

The feed intake and growth rates obtained in our trials were in general lower compared to performance experiments, as it was expected. It should be noted that fish sizes differ among experiments, which in turn directly affects their intake requirements and growth rates. Moreover, the diets were not formulated with the goal of optimizing fish growth but rather to study fish behaviour. Indeed, experiments on dietary selection do not necessarily correlate the most selected diet with optimal performance (Fortes-Silva et al. 2012; Gélineau et al. 1998; Montoya et al. 2011; Santos et al. 2019; Tidwell et al. 1991). Moreover, a lower fish performance can be related with the adaptation of fish to use self-feeders, where some animals in the same group may better assimilate the self-feeding system than others (Ferrari et al., 2014; Tidwell et al. 1991). Even in growth experiments, although a diet is formulated to provide maximum performance, when given the opportunity, fish might not prefer that formula and reduce their intake (de la Higuera 2001). Nevertheless, the lower performance indicators obtained in our experiments were not a concern, especially as no mortality occurred and fish still gained weight.

## Conclusions

The main purpose of this research was to assess the feeding behaviour and ability of Nile tilapia and gilthead seabream to self-select their preferred diets. In one hand, tilapia was able to show a preference and selected one of the given feeds by sensing its orosensory properties and formulation and based on post-ingestion and absorption signals, confirming their ability to choose a specific feed. On the other hand, gilthead seabream did not show a consistent preference for any diet. Accordingly, self-selection studies based on fish “nutritional wisdom” allow fish to exhibit their behaviour; thus, they may be considered in the initial screening of potential new aquaculture feeds, with alternative ingredients before being used commercially.

## Data Availability

The datasets generated during and/or analysed during the current study are not publicly available due to privacy concerns but are available from the corresponding author on reasonable request.
